# Single- and Dual-Source CT Myelography: Comparison of Radiation Exposure and Establishment of Diagnostic Reference Levels

**DOI:** 10.3390/diagnostics11101809

**Published:** 2021-09-30

**Authors:** Sebastian Zensen, Denise Bos, Marcel Opitz, Michael Forsting, Nika Guberina, Cornelius Deuschl, Axel Wetter

**Affiliations:** 1 Institute of Diagnostic and Interventional Radiology and Neuroradiology, University Hospital Essen, 45147 Essen, Germany; denise.bos@uk-essen.de (D.B.); marcel.opitz@uk-essen.de (M.O.); michael.forsting@uk-essen.de (M.F.); cornelius.deuschl@uk-essen.de (C.D.); axel.wetter@uk-essen.de (A.W.); 2 Department of Radiotherapy, University Hospital Essen, 45147 Essen, Germany; nika.guberina@uk-essen.de; 3 Department of Diagnostic and Interventional Radiology, Neuroradiology, Asklepios Klinikum Harburg, 21075 Hamburg, Germany

**Keywords:** radiation exposure, myelography, computed tomography, diagnostic reference level, image quality

## Abstract

CT myelography (CTM) is a diagnostic technique for the evaluation of various spinal pathologies, and plays an important role in diagnosis of different diseases such as spontaneous intracranial hypotension and postoperative cerebrospinal fluid leaks. The aims of this study were to examine radiation exposure, establish diagnostic reference levels (DRLs) and compare radiation doses of single- and dual-source examinations and different CTM protocols. In this retrospective study, 183 CTMs comprising 155 single-source and 28 dual-source examinations, performed between May 2015 and December 2020, were analyzed. Dose data included 31 whole spine (A), 23 cervical (B), 10 thoracic (C), and 119 lumbar (D) CTMs. Radiation exposure was reported for volume-weighted CT dose index (CTDI_vol_) and dose-length product (DLP). Radiation doses for CTDI_vol_ and DLP were distributed as follows (median, IQR): A: 7.44 mGy (6.01–11.17 mGy)/509.7 mGy·cm (382.4–682.9 mGy·cm), B: 9.31 mGy (7.20–14.64 mGy)/214.5 mGy·cm (153.7–308.2 mGy·cm), C: 6.80 mGy (6.14–8.26 mGy)/365.4 mGy·cm (222.8–432.4 mGy·cm), D: 11.02 mGy (7.97–14.89 mGy)/308.0 mGy·cm (224.7–413.7 mGy·cm). Local DRLs could be depicted as follows (CTDI_vol_/DLP): A: 11 mGy/683 mGy·cm, B: 15 mGy/308 mGy·cm, C: 8 mGy/432 mGy·cm, D: 15 mGy/414 mGy·cm. High image quality was achieved for all anatomical regions. Basically, radiation exposure of CTM differs according to anatomical location.

## 1. Introduction

CT myelography (CTM) is a diagnostic CT examination with prior intrathecal contrast agent administration. CTM enables the evaluation of various spinal pathologies that contact, displace, or impinge the thecal sac, cord, or nerve roots [1,2,3]. It plays an important role in several indications such as intradural extramedullary cysts, spontaneous intracranial hypotension and postoperative cerebrospinal fluid (CSF) leaks, nerve root avulsion, spinal canal stenosis, arthritis, and other degenerative and meningeal conditions [2,3]. Though the role of MRI for spinal diseases has increased continuously in recent decades due to its non-invasiveness and excellent soft tissue contrast, CTM is still indispensable, in particular when MRI is non-diagnostic or contraindicated [1,3,4]. Moreover, CTM offers advantages over MRI due to the possibility of dynamic imaging as well as different patient positioning [2,5]. In addition to the commonly used single-source CTM, examinations in dual-source mode may be useful to reduce artifacts, especially in patients with metal implants [6]. However, CT entails an inevitable radiation burden and is considered as a high-dose imaging technique since its establishment accounts for the major part of the collective effective dose for all radiographic examinations [7,8]. While dose assessment optimizes radiation protection, studies reporting radiation doses of CTM are rare and dose data differentiated by anatomical regions for CTMs of parts of the spine are lacking [9,10,11]. For various indications, diagnostic reference levels (DRLs) were established to limit radiation exposure of radiological imaging modalities, which are set at the 75th percentiles of dose metric distributions [12,13]. Though European and national DRLs are set for CT of cervical and lumbar spine, specific values for CTM are not established [12,14]. To monitor radiation dose, the volume-weighted CT dose index (CTDI_vol_) indicates the average amount of radiation exposure emitted by the CT scanner that a patient receives, whereas the dose-length product (DLP) quantifies the total amount of ionizing radiation [15,16,17]. Modern CT scanners index and archive these reference parameters, which can help ensure optimal radiation exposure, although they do not directly represent the dose for an individual patient [18].

The purpose of this study was to evaluate the radiation exposure and image quality of CTM differentiated by anatomical region, establish local DRLs and compare radiation doses of single- and dual-source examinations.

## 2. Materials and Methods

### 2.1. Patient Cohort

Between May 2015 and December 2020, dose data of all consecutive fluoroscopic and CT myelography examinations at our center were included. Patients were identified using the radiological information system (RIS) and all datasets, which provided full information for dose metrics and precisely reported anatomical location, were eligible for analysis. Clinical information was extracted from the report archived in the RIS. Ethical approval for this retrospective single-center study was granted by the institutional review board and the requirement to obtain informed consent was waived (20-9597-BO).

### 2.2. CT Myelography and CT Scanners

**Figure 1:**
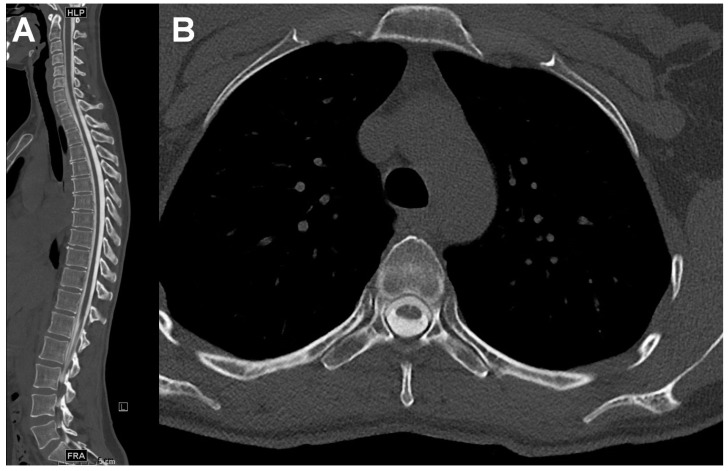
Image examples of a CT myelography of the whole spine of a 36-year-old female patient with suspected cerebrospinal fluid (CSF) leak in sagittal (**A**) and axial (**B**) reconstruction at level Th4.

**Figure 2:**
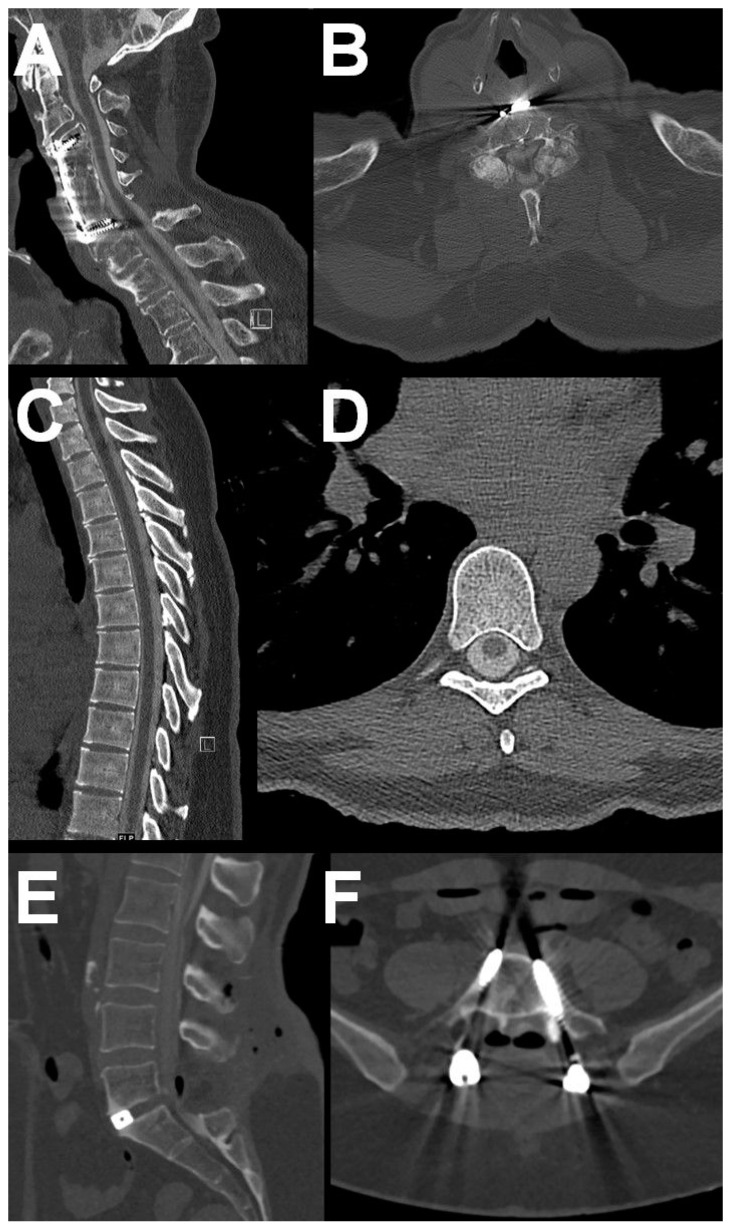
Image examples of CT myelography (CTM) of specific parts of the spine on the different CT scanners. (**A**) + (**B**): CTM on the SOMATOM Definition Flash of the cervical spine of a 79-year-old patient after ventral fusion C3–6 with impaired fine motor function of both hands with high-grade stenosis of neuroforamina C3 and C4 in the presence of marked hypertrophic spondylo-arthrosis. (**C**) + (**D**): Inconspicuous CTM on the SOMATOM Definition AS+ of the thoracic spine in a 41-year-old female patient with suspected thoracic CSF cyst. (**E**) + (**F**): CTM in dual-source technique on the SOMATOM Force of the lumbar spine of a 44-year-old female patient with a CSF leak at the level of L5 on the right after dorsal spondylosis and insertion of a disc cage. All scanners: Siemens Healthineers, Forchheim, Germany.

First, a lumbar puncture was performed under fluoroscopy, where a CSF sample was taken for cytological and clinical chemistry diagnosis. Immediately afterwards, between 8 and 20 mL of contrast medium, depending on the patient’s constitution as well as the planned myelogram type, with 250 mg iodine per ml was applied intrathecally (Solutrast 250 M, Bracco Imaging, Milan, Italy). After fluoroscopic and radiographic documentation, homogeneous intrathecal contrast medium distribution was aimed for by regular movement of the patient. CTM was then performed after approximately 30 min. CT scans were obtained at one of three commercially available, modern multi-slice CT scanners: single-source 128-slice SOMATOM Definition AS+, dual-source 128-slice SOMATOM Definition Flash and dual-source 192-slice SOMATOM Force (all Siemens Healthineers, Forchheim, Germany). At SOMATOM Force, dual-source mode was performed in patients with metal implants to reduce beam hardening artefacts. Automatic tube current modulation (CARE Dose 4D, Siemens Healthineers, Forchheim, Germany) was applied on all CT scanners. Technical settings according to scanner are shown in Table 1. The following subgroups of CTM were set: whole spine CTM and CTM of specified parts of the spine, cervical, thoracic and lumbar. Image examples of CT myelography of the whole spine and specific parts of the spine are shown in Figure 1 and Figure 2, respectively.

### 2.3. Dose Assessment

For dose assessment, examination data and dose measurements were extracted from the Digital Imaging and Communications in Medicine (DICOM) header and from the Radiation Dose Structured Report stored in the Picture Archiving and Communication System (PACS). CT dose assessments referred to the 32 cm diameter standard polymethyl methacrylate (PMMA) CT dosimetry phantom. Assessed radiation exposure indices were the CTDI_vol_ and DLP. Dose variation due to automatic tube current modulation was taken into account in the calculation of radiation exposure parameters by the CT scanners. Topogram- and monitoring-based radiation exposure data were excluded. DRLs were set at the 75th percentile of dose distribution.

### 2.4. Image Quality Assessment

Quantitative image quality analysis was performed on all CT scans by calculating signal-to-noise ratios (SNR) and contrast-to-noise ratios (CNR) according to a uniform procedure: the mean signal intensity and its standard deviation (SD) in Hounsfield units were measured in a region of interest (ROI) in the contrasted spinal canal (ROI_spinal_) and as reference tissue in the autochthonous back muscles (ROI_muscle_). Measurements were made at level C3 for CTMs of the cervical spine, at level Th6 for the thoracic spine, and at level L3 for the lumbar spine. SNR was calculated as the quotient of the signal intensity of the ROI_spinal_ and its SD. CNR was defined as the quotient of the difference between the signal intensities of ROI_spinal_ and ROI_muscle_ and SD of ROI_muscle_. SNR and CNR were calculated for the different anatomic locations and in relation to the protocols used on the CT scanners.

### 2.5. Statistical Analysis

Statistical analysis was performed using GraphPad Prism 5.01 (GraphPad Software, San Diego, CA, USA). To determine normal distribution Kolmogorov-Smirnov, Shapiro-Wilk and D’Agostino-Pearson test were applied. Normally distributed data are reported as mean ± SD, non-normally distributed data as median and interquartile range (IQR). Mann-Whitney U test was applied to compare radiation indices between single- and dual-source CTMs. Kruskal-Wallis test with Dunn-Bonferroni post-hoc test was performed to compare the radiation exposures of the different protocols of CTM of the lumbar spine and the image quality data of the different anatomic locations and at the different CT scanners. A *p*-value lower than 0.05 was considered statistically significant.

## 3. Results

### 3.1. Patient Cohort

In our retrospective study, 183 CTMs of 177 patients, comprising 50% females (88 out of 177) and 50% males (89 out of 177), who underwent CTM between May 2015 and December 2020, were eligible for evaluation. Mean age was 63.5 years (SD 15.2). Included datasets comprised a total of 31 whole spine (A), 23 cervical (B), 10 thoracic (C), and 119 lumbar (D) CTMs.

### 3.2. Radiation Exposure and DRLs

24% (44 out of 183) of all CT scans were performed at SOMATOM Definition AS+, 8% (15 out of 183) at SOMATOM Definition Flash and 68% (124 out of 183) at SOMATOM Force. Radiation exposure of CTM in terms of CTDI_vol_ and DLP was distributed as follows (median, IQR): A: 7.44 mGy (6.01–11.17 mGy)/509.7 mGy·cm (382.4–682.9 mGy·cm), B: 9.31 mGy (7.20–14.64 mGy)/214.5 mGy·cm (153.7–308.2 mGy·cm), C: 6.80 mGy (6.14–8.26 mGy)/365.4 mGy·cm (222.8–432.4 mGy·cm), D: 11.02 mGy (7.97–14.89 mGy)/308.0 mGy·cm (224.7–413.7 mGy·cm). Detailed results differentiated by CT scanner are shown in Table 2 for CTDI_vol_ and in Table 3 for DLP. Local DRLs for CTM could be depicted as follows (CTDI_vol_/DLP): A: 11 mGy/683 mGy·cm, B: 15 mGy/308 mGy·cm, C: 8 mGy/432 mGy·cm, D: 15 mGy/414 mGy·cm.

### 3.3. Comparison of Radiation Exposure of Single- and Dual-Source CT Myelography

**Figure 3:**
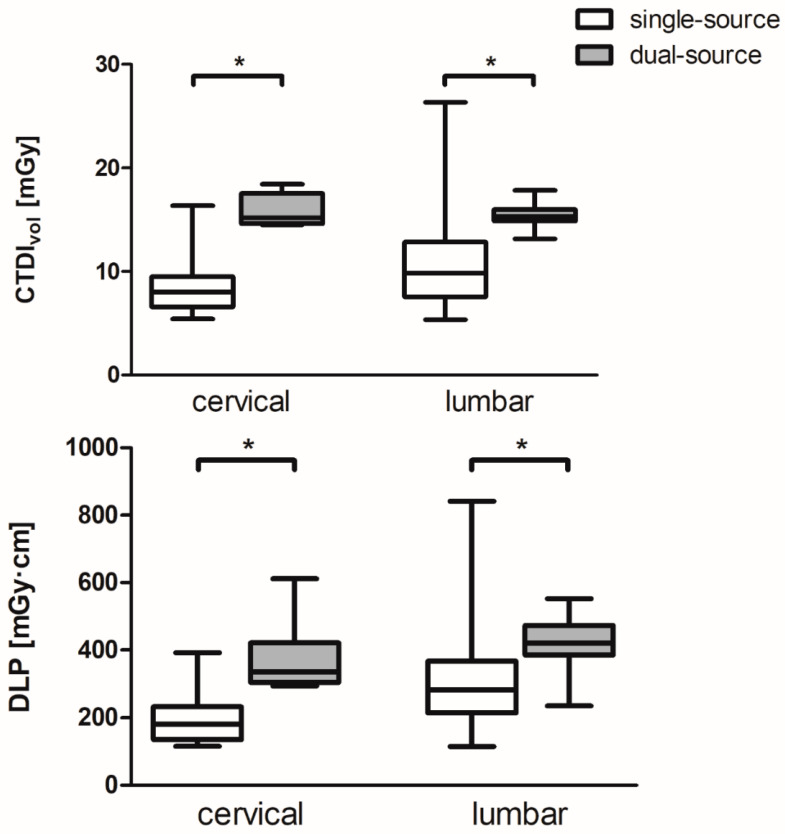
Comparison of radiation exposure in terms of volume-weighted CT dose index (CTDI_vol_) and dose-length product (DLP) for single- and dual-source CT myelography of the cervical and lumbar spine. An asterisk (*) indicates a significant difference.

Radiation exposure of single- and dual-source mode could be analyzed for cervical and lumbar spine CTMs. All dual-source CTMs were performed at SOMATOM Force. Median and IQR in terms of CTDI_vol_ and DLP were for B single-source 8.02 mGy (6.6–9.53 mGy)/181.0 mGy·cm (135.8–232.8 mGy·cm) versus dual-source 15.17 mGy (14.61–17.53 mGy)/336.6 mGy·cm (304.8–420.9 mGy·cm) and for D single-source 9.83 mGy (7.58–12.84 mGy)/282.1 mGy·cm (214.7–366.7 mGy·cm) versus dual-source 15.33 mGy (14.87–15.98 mGy)/420.6 mGy·cm (385.8–472.6 mGy·cm). Statistical analysis revealed significant higher radiation exposure for the dual-source mode in terms of CTDI_vol_ and DLP for the cervical and lumbar CTMs (all *p* < 0.001). Thus, CTDI_vol_ and DLP for cervical CTM were approximately 1.9 times higher in dual-source mode, and for lumbar CTM, CTDI_vol_ was approximately 1.6 times higher and DLP was 1.5 times higher, compared with single-source mode (Figure 3).

### 3.4. Dose Differences of CT Myelography Protocols for the Lumbar Spine

**Figure 4:**
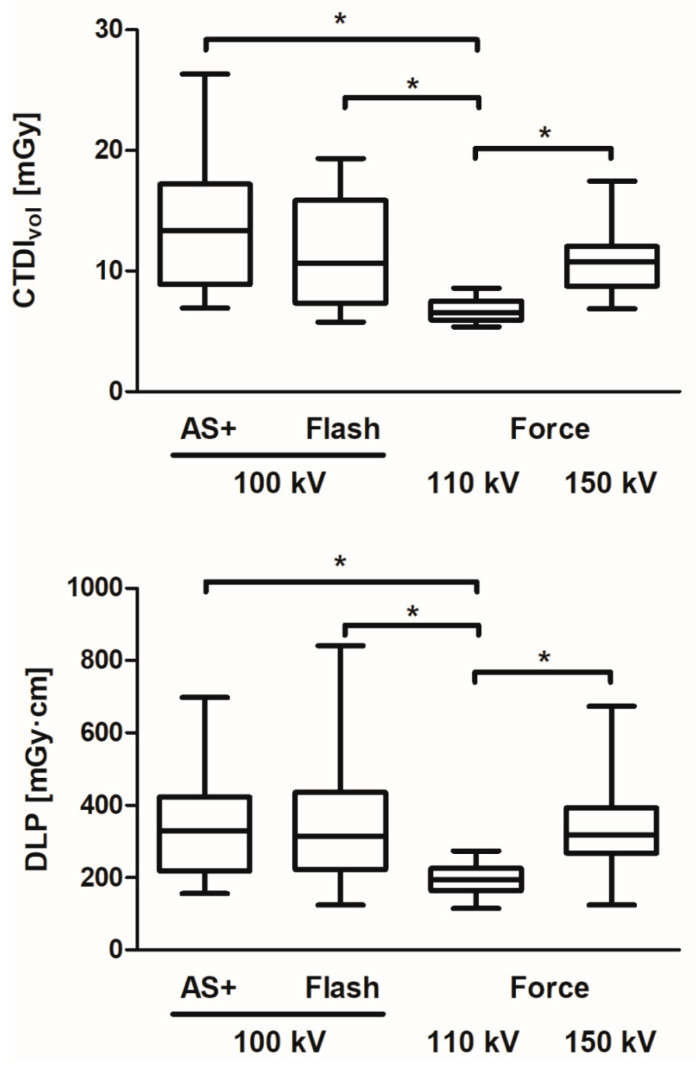
Comparison of radiation exposure in terms of volume-weighted CT dose index (CTDIvol) and dose-length product (DLP) of different CT myelography protocols for the lumbar spine on different CT scanners. AS+: SOMATOM Definition AS+; Flash: SOMATOM Definition Flash; Force: SOMATOM Force (all: Siemens Healthineers, Forchheim, Germany). Indication of tube voltage in kV refers to the applied protocols (Table 1). An asterisk (*) indicates a significant difference.

Because approximately two-thirds of all CTMs were limited to the lumbar spine, and different protocols were used here on the CT scanners (Table 1), these were examined for dose differences. Kruskal-Wallis test with Dunn-Bonferroni post hoc test showed that there were significant differences between the protocols at the different scanners for both CTDI_vol_ and DLP (all *p* < 0.001). Radiation exposure was significantly decreased for both dose descriptors at the third-generation dual-source CT SOMATOM Force with the 110 kV protocol compared to all other protocols with a median CTDI_vol_ of 6.54 mGy (IQR 5.95–7.52) and DLP of 194.4 mGy·cm (IQR 164.6–225.7) (Figure 4). In contrast, the 100 kV protocols on SOMATOM Definition AS+ and Flash showed comparable radiation exposure values with a median CTDI_vol_ of 13.35 mGy (IQR 8.88–17.20) and 10.67 mGy (IQR 7.33–15.85), respectively, and DLP of 329.1 mGy·cm (IQR 219.2–423.2) and 313.6 mGy·cm (IQR 223.1–436.5), respectively. Radiation exposure at SOMATOM Force with a higher tube voltage of 150 kV was also similar, with a median CTDI_vol_ of 10.74 mGy (IQR 8.74–12.03) and DLP of 317.8 mGy·cm (IQR 268.2–393.0), respectively.

### 3.5. Image Quality of CT Myelography

Image quality showed high SNR and CNR values for CTMs of all regions, which tended to be highest for whole spine CTM (Table 4), although statistical analysis showed no significant difference (*p* = 0.26 and *p* = 0.35, respectively). No significant difference was found in the SNR values of the CTMs on the different CT scanners (*p* = 0.29), although the 110 kV protocol on SOMATOM Force tended to have the highest SNR values (Table 4). CNR values were significantly different on the different CT scanners (*p* < 0.001). Dunn-Bonferroni post-hoc test as well as Mann-Whitney U test showed that the CNR values of the CTMs with the 110 kV protocol on the SOMATOM Force were significantly higher than for the CTMs on the SOMATOM Definition AS+ and Flash (*p* < 0.001 and *p* = 0.004, respectively). In contrast, the other CNR values on the SOMATOM Force and the other scanners were not significantly different.

## 4. Discussion

Our study analyzes radiation exposure of CTMs and reveals useful dose data differentiated by anatomical location for partial CTM. Our local DRLs may help as a benchmark to optimize radiation protection as neither international nor national DRLs are established for this examination technique.

Assessing and monitoring dose data of CT examinations helps to ensure radiation protection and optimize CT protocols [19]. Therefore, standardized collection of radiation dose data is common practice at our institute. In the recent literature, studies reporting radiation exposure of CTM are rare. Among the few studies considering dose aspects, Nicholson et al. reported a median CTDI_vol_ of about 38 mGy (range 10–104 mGy) and DLP of about 1185 mGy·cm (range 186–4849 mGy·cm) [9]. Another study reported an invariable CTDI_vol_ of 21.4 mGy and mean effective dose of 70.6 mSv (range 21.5–182.9 mSv) [11]. Not least, Dobrocky et al. depicted a mean CTDI_vol_ of 107 mGy (range 12–246 mGy) and DLP of 1347 mGy·cm (range 550–3750 mGy·cm) [10]. In comparison with these studies, our locally determined radiation exposures were remarkably lower with a median CTDI_vol_ of 7.44 mGy (range 4.25–16.15 mGy) and DLP of 509.7 mGy·cm (279.6–1033.0 mGy·cm). The decreased radiation exposure compared to these studies can be explained by modified protocol settings such as decreased tube voltage and reference tube current-time product. To our knowledge, studies reporting specific dose data of CTM differentiated by anatomical location for CTMs of parts of the spine are lacking in the recent literature. Therefore, we reported specific values for the different parts of the spine. 

Total radiation exposure in terms of DLP of partial CTMs of all regions were of course lower compared with whole spine CTM. Dose reductions attributed to performing CTM of parts of the spine compared with imaging of the entire spine range from 28% for thoracic spine to 35% for lumbar spine and up to 56% for CTM of the cervical spine when comparing median DLPs (Table 2). Therefore, critical indication of whole spine CTM for expected focal spinal pathology in distinct parts of the spine should be recommended.

CTM in dual-source mode is often preferred, especially in patients with postoperative status and metal implants after osteosynthesis, because it allows better beam hardening artifact suppression [6,20,21]. In the literature, studies on the impact of the dual-source mode on radiation exposure of CT are divergent [22]: our results, like other studies [23,24,25], show a slight dose increase, although in principle a comparable radiation exposure can also be achieved [26,27]. Radiation exposure on the SOMATOM Force was slightly lower in dual-source mode for CTM compared with the study of Grams et al. [6].

Comparison of the different protocols for the lumbar spine on the CT scanners showed that the 110 kV protocol on the SOMATOM Force required significantly lower radiation exposure compared with all other protocols. Compared to the highest radiation exposure for lumbar CTM with the 100 kV protocol on the SOMATOM Definition AS+, the radiation exposure here was about half as high in terms of CTDIvol and as much as 40% lower in terms of DLP. Compared to the radiation exposure at SOMATOM Definition Flash with the 100 kV protocol, which was very similar to that of the 150 kV protocol at SOMATOM Force, the dose reduction of the 110 kV protocol at SOMATOM Force was about 40%. Compared to the protocols with 100 kV, the significant dose reduction of up to 50% is mainly explained by the lower reference effective tube current time product. However, when compared also to the 150 kV protocol, which had an even lower reference effective tube current time product, the radiation exposure was reduced by about 40%, which can be attributed to the lower tube voltage.

A decisive criterion when performing a CT examination is that the image quality is sufficient for diagnostics. A dose reduction that leads to insufficient image quality and, if necessary, makes it necessary to repeat the examination contradicts the principle of radiation protection. All examinations were from routine clinical practice and our data show that despite the low radiation exposure, CTMs can be acquired at all anatomical locations with high objective image quality, which was not significant but tended to be highest for imaging of the whole spine. Among the different protocols, the 110 kV protocol on the SOMATOM Force provided the highest image quality, which was significantly increased to the scans on SOMATOM Definition AS+ and Flash, but comparable to the other protocols on the same scanner. In CTM, contrasting the subarachnoid space with the contrast agent previously injected under fluoroscopy is critical to adequately assess CTM questions usually related to CSF distribution or possible leakage. Since the objective image quality parameters based on contrasting the subarachnoid space thus also depend on the distribution and presumably applied amount of the injected contrast agent, a detailed investigation of the influence on image quality and the determination of optimal contrast agent amounts would be helpful in further studies.

A helpful benchmark for dose protection approaches are DRLs which indicate typical ionizing radiation exposure values in a country, region or an institute [28]. Though European and national DRLs are established for CT of cervical and lumbar spine, specific values for CTM are lacking. A European DRL is only established for lumbar spine CT (CTDI_vol_ 35 mGy, range 30–55 mGy; DLP 500 mGy·cm, range 300–870 mGy·cm) without consideration of CTM [12]. Yet, our local DRLs undercut those values of 36 European countries plainly. German DRLs are defined for the cervical and lumbar spine, differentiated according to whether they are aimed at imaging the intervertebral disc (cervical and lumbar: CTDI_vol_ 25 mGy) or the bone (cervical spine: 20 mGy, lumbar spine: 10 mGy) [14]. Our local DRLs of CTM were well below national DRLs for both cervical and lumbar spine, but for the reference levels for lumbar spine bone imaging, the local DRLs were higher. Hence, completed DRLs might be another essential step towards radiation protection and dose optimization, and our locally established DRLs may help as benchmarks as they were well below the recently published dose data of CTMs. Furthermore, to our knowledge, this is the first time that DRLs have been reported for CTMs of parts of the spine.

Limitations of our study are the retrospective design and that there were no equivalent numbers of CTMs at different regions and CT scanners and single- and dual-source CT examinations. Although our results tended to show higher radiation exposures for CTMs in dual-source mode, further analysis is still needed to draw accurate conclusions about dose differences due to the retrospective study design and different protocols. Furthermore, due to the small size of some subgroups of the patient cohort, it is recommended to investigate radiation exposure in a larger population in multicenter studies, which could be the next necessary step for setting national and European DRLs. Strengths of our study include the combination of data sets from single- and dual-source CT examinations, including image quality assessment differentiated by anatomical locations, which enables specific dose assessment.

## 5. Conclusions

In conclusion, specific radiation exposure values for CTM, differentiated by anatomical location, were determined. Despite low radiation exposure, CTMs with high image quality can be acquired, and dose reduction may be possible, particularly by adjusting the scan parameters for the lumbar spine. As DRLs for CTM are needed to optimize radiation protection, we reported detailed, locally determined DRLs, which may serve as benchmarks.

## Figures and Tables

**Table 1:** Technical parameters of CT myelography at three different multi-slice CT scanners.

CT Scanner	Siemens SOMATOM Definition AS+	Siemens SOMATOM Definition Flash	Siemens SOMATOM Force ^1^		
Collimation	128 × 0.6 mm	128 × 0.6 mm	192 × 0.6 mm		
No. of examinations	44	15	48	48	28
Dual-source mode	N/A	off	off	off	on
Tube voltage (kV)	100	100	110	150	100/Sn150 ^2^
Reference effective tube current-time product (mAs)	316	273	171	81	190/380
Automatic tube current modulation	on	on	on	on	on
Rotation time (s)	1	1	1	1	0.5

^1^ Several protocols with different settings were used on this scanner. ^2^ At tube B tin (Sn) filtration was applied.

**Table 2:** Volume-weighted CT dose index (CTDI_vol_) of CT myelography.

			CTDI_vol_ [mGy]						
Anatomical Location	CT Scanner *	No. of Scans	Min	25th Percentile	Median	75th Percentile	Max	Mean	SD
(A) Whole spine	Total	31	4.25	6.01	7.44	11.17	16.15	8.81	3.75
	AS+	4	10.13	11.16	14.84	15.97	16.15	13.99	2.69
	Flash	1	-	-	7.97	-	-	-	-
	Force	26	4.25	5.67	6.91	10.02	15.74	8.04	3.32
(B) Cervical spine	Total	23	5.42	7.20	9.31	14.64	18.44	10.44	4.05
	AS+	4	7.57	7.96	9.85	10.66	10.69	9.49	1.47
	Flash	2	9.31	-	12.84	-	16.36	12.84	4.99
	Force	17	5.42	6.60	8.88	14.76	18.44	10.38	4.43
(C) Thoracic spine	Total	10	5.66	6.14	6.80	8.26	24.58	8.74	5.72
	AS+	3	5.66	-	10.31	-	24.58	13.52	9.86
	Flash	0	-	-	-	-	-	-	-
	Force	7	5.75	6.27	6.60	7.25	7.58	6.69	0.63
(D) Lumbar spine	Total	119	5.36	7.97	11.02	14.89	26.34	11.52	4.37
	AS+	33	6.91	8.88	13.35	17.20	26.34	13.63	5.15
	Flash	12	5.75	7.33	10.67	15.85	19.34	11.34	4.68
	Force	74	5.36	7.45	10.09	13.53	17.83	10.62	3.62

* AS+: SOMATOM Definition AS+; Flash: SOMATOM Definition Flash; Force: SOMATOM Force (all: Siemens Healthineers, Forchheim, Germany).

**Table 3:** Dose-length product (DLP) of CT myelography.

			DLP [mGy·cm]						
Anatomical Location	CT Scanner *	No. of Scans	Min	25th Percentile	Median	75th Percentile	Max	Mean	SD
(A) Whole spine	Total	31	279.6	382.4	509.7	682.9	1033.0	542.9	199.1
	AS+	4	530.5	606.5	893.9	1013.0	1033.0	837.8	220.5
	Flash	1	-	-	530.2	-	-	-	-
	Force	26	279.6	367.7	481.6	583.6	850.6	498.1	160.4
(B) Cervical spine	Total	23	115.5	153.7	214.5	308.2	611.5	241.5	115.4
	AS+	4	137.6	150.8	209.1	275.7	291.6	211.9	64.8
	Flash	2	237.5	-	314.8	-	392.1	314.8	109.3
	Force	17	115.5	143.9	185.9	312.1	611.5	239.9	126.0
(C) Thoracic spine	Total	10	123.8	222.8	365.4	432.4	500.8	340.1	119.8
	AS+	3	123.8	-	416.3	-	500.8	347.0	197.8
	Flash	0	-	-	-	-	-	-	-
	Force	7	199.2	230.6	356.3	431.6	434.7	337.1	91.83
(D) Lumbar spine	Total	119	114.3	224.7	308.0	413.7	841.5	325.9	134.2
	AS+	33	156.1	219.2	329.1	423.2	698.0	337.2	136.8
	Flash	12	124.8	223.1	313.6	436.5	841.5	353.2	193.0
	Force	74	114.3	224.0	303.8	404.9	674.1	316.4	122.6

* AS+: SOMATOM Definition AS+; Flash: SOMATOM Definition Flash; Force: SOMATOM Force (all: Siemens Healthineers, Forchheim, Germany).

**Table 4:** Quantitative image quality analysis of CT myelography.

		Signal-to-Noise Ratio (SNR)		Contrast-to-Noise Ratio (CNR)	
		Median	IQR	Median	IQR
Anatomical location					
(A) Whole spine (B) Cervical spine (C) Thoracic spine (D) Lumbar spine		40	34	80	148
		25	46	54	50
		25	49	57	120
		26	23	48	58
CT scanner *					
AS+ Flash Force (110 kV protocol) Force (150 kV protocol) Force (100/Sn150 kV protocol)		26	26	33	31
		23	19	21	20
		34	38	74	57
		24	24	51	66
		27	17	50	46

* AS+: SOMATOM Definition AS+; Flash: SOMATOM Definition Flash; Force: SOMATOM Force (all: Siemens Healthineers, Forchheim, Germany).
